# Successful resection of liver metastasis detected by exacerbation of skin symptom in a patient with dermatomyositis accompanied by rectal cancer: a case report and literature review

**DOI:** 10.1186/s40792-016-0281-z

**Published:** 2017-01-04

**Authors:** Kosuke Ono, Manabu Shimomura, Kazuhiro Toyota, Atsushi Kagimoto, Naofumi Tsukiyama, Masayuki Shishida, Koichi Oishi, Kazuaki Miyamoto, Satoshi Shibata, Masahiro Ikeda, Seiji Sadamoto, Tadateru Takahashi

**Affiliations:** 1 Department of Surgery, National Hospital Organization Higashihiroshima Medical Center, 513, Jike, Saijyo-cho, Higashihiroshima, Hiroshima 739-0041 Japan; 2 Department of Gastroenterological and transplant Surgery, Applied life sciences, Institute of Biomedical & Health sciences, Hiroshima University, Hiroshima, Japan

**Keywords:** Dermatomyositis, Colorectal cancer, Recurrence, Liver metastasis

## Abstract

**Background:**

Dermatomyositis (DM) is a rare syndrome that belongs to the group of idiopathic inflammatory myopathies. The association between DM and malignancy is well recognized, and the severity of DM symptoms has been linked to the progression of metastatic disease.

**Case presentation:**

We report the case of a 42-year-old man that was diagnosed with dermatomyositis (DM) and rectal cancer. Proctectomy was performed, and DM symptoms were resolved postoperatively. One year and 9 months after the surgery, liver metastasis occurred accompanied by the exacerbation of DM symptom. Partial resection of the liver was performed, and postoperative course was uneventful. DM symptoms improved postoperatively, and no evidence of cancer recurrence or DM symptoms was observed 2 years after the second surgery. To date, few reports have described recurring cases of DM accompanied by colorectal cancer in detail. We reviewed four similar cases that were reported poor prognoses with treatment resistance. However, our case report demonstrates good long-term results with resection of metastatic lesion.

**Conclusions:**

It is important to check the exacerbation of DM symptoms, as this symptom sometimes preceded cancer relapse during the follow-up of our patient with DM and colorectal cancer.

## Background

Dermatomyositis (DM) is a rare syndrome that belongs to the group of idiopathic inflammatory myopathies. It is characterized by heliotrope rashes, Gottron’s papules, and proximal muscle weakness [1, 2]. The association between DM and malignancy has been well recognized for many years [3]. In particular, DM has been linked to tumors in the ovaries, lungs, pancreas, and stomach; as well as non-Hodgkin’s lymphoma, and sometimes, colorectal cancer [4]. Malignancies are usually diagnosed within a year from the onset of myopathy, and the extent of DM symptoms has been associated with the progression of metastatic diseases [5]. Recent reports have demonstrated the relationship between the exacerbation of DM symptoms and cancer recurrence [6].

We report a case of a patient who presented with DM and colorectal cancer, and underwent curative resection for liver metastasis that was detected by the exacerbation of DM symptom. We also reviewed similar reported cases and analyzed the clinicopathological features of patients with DM accompanied by recurrent colorectal cancer.

## Case presentation

**Fig. 1:**
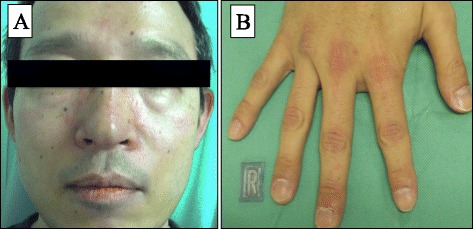
**a** Facial erythma and heliotrope rash. **b** Gottron’s papules overlying knuckles

**Fig. 2:**
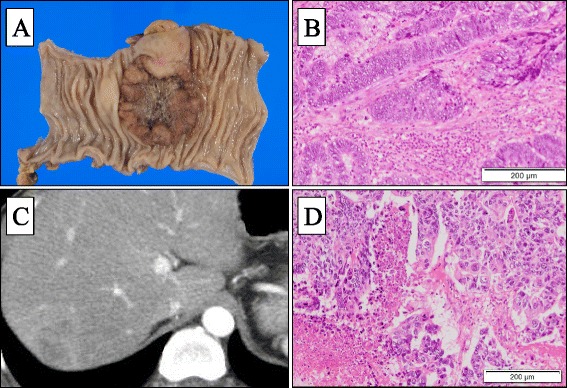
**a** 4.7 cm × 4.6 cm-sized type 2 tumor in the rectosigmoid colon. **b** The pathological diagnosis was moderately differented adenocarcinoma (HE staining). **c** CT scan revealed a 30-mm tumor in segment 7 of the liver. **d** Metastatic adenocarcinoma was confirmed in the histopathological examination

A 42-year-old man presented with a skin rash on his face and finger (Fig. 1). His past history included an appendectomy at 15 years old. There was no family history. The patient was diagnosed with DM based on the presence of a heliotrope rash, Gottron’s papules, dysphagia, proximal muscle weakness, and raised muscle-associated enzyme levels; Jo-1 antibody was negative. He underwent medical examinations to evaluate the existence of accompanying malignant tumors. Colonoscopy revealed type 2 tumors in the rectum (Fig. 2a); the patient was diagnosed with adenocarcinoma following a biopsy. Computed tomography (CT) scan showed no distant metastases and some swollen lymph nodes. We performed anterior resection of the rectum and regional lymphadenectomy. The pathological diagnosis was moderately differentiated adenocarcinoma, T3N1M0, and stage III according to the 7th edition of the International Union Against Cancer TNM classification (Fig. 2b). DM symptoms improved after surgery. The patient received adjuvant chemotherapy of capecitabine for 6 months, and blood examinations were performed every 3 months during follow-up at the outpatient clinic. No evidence of the recurrence in the tumor marker, CT scan, and colonoscopy were observed 1 year postoperatively. During this period, he did not receive immunosuppressants such as predonisolone.

One year and 9 months after surgery, he was referred to the hospital due to exacerbated skin symptoms, including heliotrope rash and Gottron’s papules. The laboratory examination revealed elevated levels of creatine kinase (671 U/l), aspartate transaminase (122 IU/l), lactate dehydrogenase (525 IU/l), and carcinoembryonic antigens (6.5 ng/ml). Based on these findings, we suspected rectal cancer recurrence, and the CT scan revealed a 30-mm tumor in the segment 7 of the liver (Fig. 2c). There were no other distant metastases in the gastrointestinal endoscopy and colonoscopy. The patient was diagnosed with recurring rectal cancer, and he underwent partial resection of the liver at segment 7. Metastatic adenocarcinoma was confirmed in the histopathological examination (Fig. 2d); postoperative course was uneventful.

The patient’s skin symptoms improved postoperatively. Laboratory values including CPK, aldolase, and CEA also decreased. Capecitabine was administrated as an adjuvant chemotherapy for 6 months. No evidence of cancer recurrence and DM symptoms were observed 2 years after the hepatectomy.

## Discussion

The association between DM and malignant tumors was independently described nearly 100 years ago by two investigators, Kankeleit and Stertz [7]. The incidence of cancer in adult patients with DM is approximately 13–42% [8]. Although its underlying pathogenesis remains unknown, it has been considered as one of the probable reasons why paraneoplastic condition producing bioactive mediators causes immune reactions in the muscle fibers and skin [5, 9]. Patients with DM and malignant disease have reportedly a worse prognosis than patients with idiopathic DM; surgical resection was not possible in many of the patients with cancer because of the advanced stage of their disease [10]. Difficulties in the management of cancer chemotherapy and medication for DM symptoms have also been reported due to severe dysphagia in patients with unresectable metastases [6]. Successful treatment of these malignancies can lead to resolution of paraneoplastic symptoms. Several reports have shown that tumor resection can lead to the resolution of paraneoplastic DM symptoms [11]. In our case, the extent of DM symptoms was associated with the treatment course of rectal cancer, suggesting that we were dealing with a case of paraneoplastic DM caused by rectal cancer.

It has been reported that symptoms also worsen when the malignancy recurred after resection of the primary disease in patients with paraneoplastic DM. However, to date, few reports have described details of recurring disease in the patients with DM accompanied by colorectal cancer. We have therefore investigated recurrent cases of DM accompanied by colorectal cancer to clarify the clinical features of these cases.

A literature search was conducted in April 2016 using the PubMed and Ichushi database to obtain English and Japanese literature describing recurring cases of patients with colorectal cancer and DM; four reports were found [6, 12–14].The keywords used in the search were “dermatomyositis” and “colorectal cancer.” The details of clinicopathological features of these cases, including the present case, are demonstrated in Table 1. There were two men and three women with a mean age of 54 years. Three patients had colon cancer and two had rectal cancer. Two cases had synchronous liver metastases, and hepatic resections were performed after the resection of the primary tumors. Recurrence sites were in the liver in three cases, lung in two cases, and local recurrence in one case (including overlapped case). Four reported cases presented moderately differentiated adenocarcinoma; however, the pathological differences between general colorectal cancers were not determined. The molecular findings for such cases were not described, although one case presented BRAF V600E mutation (case 3). Further investigation is needed to clarify these findings. DM symptoms worsened when cancer recurred in four cases, except in one case that was not described in detail. In the two cases including our case, cancer relapses were detected by exacerbation of DM symptoms, although these were not preceded by cancer relapses in the other two cases. Cancer recurrence occurred within 3 to 7 months after surgery in all cases, except for the case we present here. Systemic chemotherapies were applied in all four cases, and three of the four patients died from disease. Based on these findings, patients with paraneoplastic disease were suggested to have resistance to surgery, although poor prognoses compared with common colorectal cancer could not be determined because of the small sample size. The present case demonstrated good outcomes with resection of metastatic disease and relatively long disease-free survival.

**Table 1:** Reported cases

Case	Author (year)	Sex	Age	Location	Stage	Recurrence site	Histological type	Disease-free interval	DM symptom related to the recurrence	Treatment for recurrent disease	Outcome (time after recurrence)
1	Ishii S (1990)	F	53	Rectum	I	Local	Mod.	4 months	Present	Chemotherapy	DOD (2 months)
2	Yamada K (1999)	M	58	Colon	IV	Liver	Mod.	3 months	ND	Chemotherapy	DOD (2 years)
3	Landriscina M (2013)	F	71	Colon	IV	Lung	ND	7 months	Present	Chemotherapy	DOD (ND)
4	Nagano Y (2015)	F	44	Rectum	II	Lung and liver	Mod.	4 months	Present	Chemotherapy	Alive with disease (7 months)
5	Present case (2016)	M	42	Rectum	III	Liver	Mod.	21 months	Present	Resection	NED (2 years)

*DM* dermatomyositis, *Mod.* moderately differentiated adenocarcinoma, *ND* not described, *DOD* died of disease, *NED* no evidence of disease

Most patients presented with exacerbating DM symptoms when the colorectal cancer relapsed. It is important to check the exacerbation of DM symptoms, including skin erythema and dysphagia, when following patients with paraneoplastic DM. Few studies reported recurring cases of DM and other cancers. These reports demonstrated the association between DM symptoms and cancer recurrence in patients with ovarian cancer [15], breast cancer [16], and bladder cancer [17], supporting our findings in colorectal cancer. The present report describes a rare case with a good outcome of resecting liver metastases that were detected early by exacerbation of skin symptoms before annual examination in a patient with DM accompanied by rectal cancer.

## Conclusions

In conclusion, patients with paraneoplastic DM generally have poor prognoses with treatment resistance. However, the present report demonstrates that there are several cases with good, long-term results with resection of metastatic diseases. It is important to check for the exacerbation of DM symptoms because this symptom sometimes appears to precede cancer relapse in the follow-up of patients with colorectal cancer and DM.

## Abbreviations

CEA: Carcinoembryonic antigen
CT: Computed tomography
DM: Dermatomyositis
